# Fibrosis progression in paired liver biopsies from HIV/HCV co-infected patients

**Published:** 2011-07-01

**Authors:** Monica Schiavini, Elena Angeli, Annalisa Mainini, Caterina Uberti-Foppa, Pietro Zerbi, Caterina Sagnelli, Antonietta Cargnel, Gianluca Vago, Pier Giorgio Duca, Riccardo Giorgi, Giuliano Rizzardini, Guido Gubertini

**Affiliations:** 1 Department of Infectious Diseases, L. Sacco Hospital, Milan, Italy; 2 Division of Infectious Diseases, San Raffaele Scientific Institute, Milan, Italy; 3 Department of Pathology, L.Sacco Hospital, Milan, Italy; 4 AIDS-Aid Foundation, Milan, Italy; 5 Medical Statistic Unit, Preclinical Science Department, Milan University, Milan, Italy

**Keywords:** HIV, HCV, Liver fibrosis, Antiretroviral therapy

## Abstract

**Background:**

Chronic hepatitis C is more aggressive during HIV infection. Available data about risk factors of liver fibrosis in HIV/HCV co-infected patients derive from studies based on a single liver biopsy.

**Objectives:**

To evaluate the risk factors of liver fibrosis progression (LFP) and to investigate the role of antiretroviral therapy (ARV) in HIV/HCV patients who underwent paired liver biopsy.

**Patients and Methods:**

We retrospectively studied 58 patients followed at two Infectious Diseases Departments in Northern Italy during the period 1988-2005. All specimens were double-blinded and centrally examined by two pathologists. LFP was defined when an increase of at least one stage occurred in the second biopsy, according to the Ishak-Knodell classification.

**Results:**

In a univariate analysis, serum levels of alanine aminotransferase (ALT) > 150 IU/L at the first biopsy (P = 0.02), and a > 20% decrease in CD4+ cell count between the two biopsies (P = 0.007), were significantly associated with LFP. In multivariate analysis, a > 20% decrease in CD4+ cell count remained independently associated to LFP (Odds Ratio, 3.99; 95% confidence interval, 1.25-12.76; P < 0.02). Analysis of life survival curves confirmed the correlation between CD4+ cell count and LFP.

**Conclusions:**

Our findings highlight that in HIV/HCV coinfected patients, an effective antiretroviral therapy that assures a good immune-virological profile contributes to reducing the risk of LFP.

## 1. Background

The use of highly active antiretroviral therapy (HAART) has significantly improved the life expectancy of HIV-infected patients mostly due to a drop in opportunistic infections, while on the other hand the mortality rate due to liver disease has dramatically increased [1][2][3][4][5]. Due to the similar routes of transmission of HCV and HIV, the prevalence of HCV infection in HIV-positive patients ranges from 30 to 50%, and can reach up to 90% among injecting drug users [6][7][8][9]. Several studies have shown that HIV/HCV co-infected patients show a more rapid progression to cirrhosis [10][11][12]. Different factors contribute to the accelerated evolution of liver disease; central among these is HIV-induced immunosuppression. The effect of HAART on liver fibrosis remains controversial. Recent data have demonstrated that HAART is associated with a reduction of liver-related mortality in HIV/HCV co-infected patients [13][14]. A few retrospective studies have reported a relationship between regimens of antiretroviral therapy containing protease inhibitors (PI), and slower fibrosis progression [15][16][17]. However, several studies revealed no association between HAART and liver fibrosis progression [18][19][20][21]. Available data on risk factors for liver fibrosis progression in HIV/HCV co-infected patients derive mostly from retrospective studies based on a single liver biopsy and an estimated duration of HCV infection, which make the assumptions that the reported date of infection was reliable and that liver fibrosis progressed at a linear rate. Considering these limitations, we carried out a study to analyze liver fibrosis progression in HIV/HCV co-infected patients who underwent paired liver biopsies.

## 2. Objectives

The first end point of this study was to evaluate liver fibrosis progression (LFP) and its associated risk factors. LFP was defined as an increase of at least one stage at the second biopsy. The fibrosis progression rate (FPR) was defined as the difference between scores at two consecutive biopsies divided by the time in years elapsed between these two biopsies. The second end point was to evaluate the effect of antiretroviral therapy on the progression of liver fibrosis.

## 3. Patients and Methods

We retrospectively studied HIV/HCV patients who underwent paired liver biopsies during the period 1988-2005. Patients enrolled were addressed at the II Department of Infectious Diseases of L. Sacco Hospital and at the Division of Infectious Diseases, S. Raffaele Scientific Institute, Milan, Italy. HCV infection was defined by a positive serology result after a second-generation enzyme-linked immunosorbent assay (ELISA), and positivity for plasma HCV-RNA through a branched-DNA PCR assay (Bayer). Only patients who underwent two sequential liver biopsies with an adequate sample for histological analysis and who had available clinical data were included in the study.

### 3.1. Patient evaluation

For each patient a case report was recorded, including epidemiological and clinical features [sex, age, risk factors for HIV infection, HIV stage (according to 1992 revised Centers for Disease Control and Prevention (CDC) classification), and history of high alcohol intake (defined as a consumption of > 50 g of alcohol per day)], biochemical data [levels of aspartate aminotransferase (AST) and alanine aminotransferase (ALT)], immune profile (CD4+ cell count), and HIV RNA level (available from 1996), recorded at the time of the first liver biopsy and subsequently every year until the second biopsy. The study was conducted with local Ethical Committee approval and all patients signed specific consent forms.

### 3.2. Histological evaluation

Percutaneous liver biopsies were performed using Menghini's needle to obtain specimens > 10 mm long, which were fixed in 10% formalin buffer and stained with hematoxylin-eosin. The threshold of adequacy for histological assessment was the presence of more than 10 portal tracts. All specimens were double-blinded and centrally examined by two experienced pathologists, who were not aware of the clinical and biological data of the patients. The Knodell score system, modified by Ishak (1995, revised in 2000) [22][23], was used to assess necroinflammatory activity and fibrosis. Patients at stage 5-6 during the baseline liver biopsy were excluded.

### 3.3. Statistical analysis

Software packages Graph Pad Prism (version 3.02) for Windows, Graph Pad Instat (version 3.05) for Windows 95 (Graph Pad Software, San Diego, CA, USA, www.graphpad.com), and Stata 7.0 (Stata Corporation - Lakeway Drive College Station, Texas USA, www.stata.com) were used. Descriptive statistics were expressed as medians and Interquartile Ranges (IQR) or mean and 95% Confidence Interval (95% CI), and as percentage, for continuous and categorical variables, respectively. Continuous variables were compared using the nonparametric Mann-Whitney test and the unpaired t test when appropriate. Differences in proportions were performed using Fisher's exact test. Investigated risk factors for LFP included: age at liver biopsy, gender, risk factors for HIV transmission, clinical stage of the disease, baseline and nadir CD4+ cell counts, HIV RNA at baseline, modifications of CD4+ cell counts and HIV RNA (when available) between two consecutive biopsies, ALT levels, HCV genotype, daily alcohol intake, antiretroviral therapy, and histological grading of activity (according to the Knodell-Ishak score). For each parameter, univariate logistic regression analysis was performed to calculate odds ratio (OR) and 95% CI for fibrosis progression versus non-progression. Only significant variables in univariate analysis were included in multivariate analysis by means of logistic regression. The Kaplan-Meir plot (log rank test) was used to identify differences in time for LFP in relation to the trends of CD4+ cell count between the two biopsies and to the use of HAART.

## 4. Results

### 4.1. Study population

During the study period, 65 HIV/HCV co-infected patients with paired liver biopsies were considered. Among them, 58 patients met the inclusion criteria. The main characteristics of the patients at the first biopsy are shown in Table 1. The mean time between the two biopsies was 42.64 months (95% CI 33.7-51.5). Most of the patients were male, with median age of 34 years. The median of the CD4+ cell count was high (> 400/mm(3)). 6 out of 58 patients (10.3%) had CD4+ cells count < 200/mm(3) at baseline and 33 (56.8%) had CD4+ cell count < 200 cells/mm(3) at nadir. The median ALT value was 114 IU/L; 17 out of 58 patients (29.3%) had ALT values > 150 IU/L. Most of the patients had a baseline stage of ≥ 2 (51.7%). At baseline, 20 patients (34.5%) received no antiretroviral therapy, 27 patients (46.5%) received monotherapy or a combination of two nucleoside reverse transcriptase inhibitors (NRTIs) and 11 patients (19%) received HAART. In the period between the two biopsies, six patients (10%) remained naive to antiretroviral therapy, 32 patients (55%) received monotherapy or a combination of two NRTIs, and 20 patients (35%) received HAART. Forty out of 58 (69%) patients were treated with interferon therapy after the first biopsy. All patients were non-responders or prematurely discontinued anti-HCV treatment.

**Table 1:** Characteristics of the patients at first liver biopsy

**Characteristics**	
**Sex** (Male), No. (%)	43 (74)
**Age**, y (IQR a)	34 (22-39.5)
**Risk category**, No. (%)	
** IDU b**	49 (84)
** Sexual contact**	9 (16)
**CDC c stage**, No. (%)	
** A**	25 (43)
** B**	17 (29)
** C**	16 (28)
**ART d received**, No. (%)	
** None**	20 (34.5)
** Single or dual**	27 (46.5)
** HAART e**	11 (19)
**CD4+ T cells count at **	
** Liver biopsy**, cells/mmc (IQR)	449.5 (308-676)
** Nadir**, cells/mmc (IQR)	194 (97-290)
**HCV genotype**, No. (%)	
** 1**	18 (31,1)
** 2**	1 (1,7)
** 3**	21 (36,2)
** 4**	4 (6,9)
** Not available**	14 (24,1)
**Staging**, No. (%)	
** 0-1**	28(48.2)
** 2**	17(29.3)
** 3**	10(17.2)
** 4**	2 (3.3)
**History of alcohol abuse**, No. (%)	21 (36)
**ALT f at liver biopsy**, IU/l (IQR)	114 (72-163)
**Grading mean 95%CI g**	4 (3.4-4.5)
**Staging mean 95%CI**	1.67 (1.37-1.97)

^a^ IQR: interquartile range

^b^ IDU: injecting drug use

^c^ CDC: Centers for Disease Control and Prevention

^d^ ART: antiretroviral therapy

^e^ HAART: highly active antiretroviral therapy

^f^ ALT: alanine aminotransferase

^g^ CI: confidence interval

### 4.2. Liver fibrosis progression

LFP was observed in 27 patients (46.5%). 9 out of 58 patients (15.5%) showed an increase of more than two fibrosis stages in the second biopsy. Five out of 58 patients (8.6%) developed cirrhosis. The mean time between the two biopsies was comparable in the two groups: 38.69 months (95% CI 28.2-49.0) in patients who progressed versus 45.9 months (95% CI 31.6-60.2) in patients who did not (P = 0.93 Mann Whitney test). A comparison of patients who progressed and those who did not showed a significant difference in mean stage score at the first biopsy (1.3 ± 0.9 vs. 1.96 ± 1.22, P = 0.03; unpaired t test) and in mean ALT values at baseline (173.41 ± 26.52 IU/L vs. 111.55 ± 50.6 IU/L, P = 0.03; unpaired t test with Welch correction). Age, grading score, and CD4+ cell counts at baseline and at nadir were comparable between the two groups. In 25 patients out of 58 (43%) CD4+ cell count decreased by more than 20%, and in 22 patients (38%) increased by more than 20% between the two biopsies. In the group of patients who progressed, CD4+ cell count decreased by more than 20% in 17 out of 27 patients (62.9%). HIV RNA level data was available for the 20 patients who received HAART between the two biopsies. HIV RNA remained undetectable (less than the cutoff value) in most of the determinations recorded between the two biopsies in only three out of the 10 patients who progressed (30%), and in seven out 10 (70%) in the group of non-progressors.

### 4.3. Risk factors for fibrosis progression

We studied the correlation between the LFP and age, gender, route of HIV transmission, CDC stage of HIV infection, history of alcohol abuse, ALT > 150 IU/L at baseline (> grade 2, according to ACTG scale of liver toxicity), CD4+ cell count < 200/mm(3) at nadir and < 350/mm(3) at first biopsy, detectable HIV RNA during more than 50% of the time period considered (available in the patients treated with HAART between two biopsies), histological necroinflammatory index (grading), and antiretroviral therapy. The results of the univariate analysis are summarized in Table 2. A decrease of > 20% in CD4+ cell count between the two biopsies (OR 4.88, 95% CI 1.59-15, P = 0.007), and higher ALT values at first biopsy (OR 4.16, 95% CI 1.22-14.1, P = 0.02), were significantly associated with the progression of liver fibrosis. Moreover, detectable HIV RNA, high alcohol consumption, and a drop of CD4+ cell count to < 200/mm(3) between the two biopsies were also related to a high risk of progression even if they did not reach statistical significance.

**Table 2:** Univariate analysis of factors associated with liver fibrosis progression

**Factors**	**OR a, 95% CI b**	***P* value**
**Age** (>35 y at liver biopsy)	0.7 (0.24-2.04)	0.59
**Sex** (Male)	1.43 (0.43-4.72)	0.7
**History of alcohol abuse**	2.67 (0.88-8.04)	0.067
**ALT c** (>150, IU/L at first biopsy)	4.16 (1.22-14.11)	0.02
**No interferon treatment**	2.35 (0.75-7.36)	0.16
**CD4**		
** Nadir**, < 200/mmc	2.1 (0.73-6.19)	0.19
** At first biopsy**, < 350/mmc	0.69 (0.23-2.02)	0.59
** Between two biopsies**, < 200/mmc	3.37 (0.9-12)	0.06
**No ART d, Single or dual NRTIs e*vs.* HAART f**		
** At first biopsy**	0.67 (0.1-2.5)	0.73
** Between two biopsies**	0.8 (0.2-2.39)	0.7
**Decrease of CD4** (> 20% between two biopsies)	4.88 (1.59-15)	0.007
**Detectable HIVRNA**	5.44 (0.8-36.8)	0.08
**Route of HIV transmission** (IDU g*vs.* other)	1.1 (0.2-4.6)	1
**CDC h stage** (C *vs.* A or B)	1.7 (0.5-5.4)	0.3

^a^ OR: Odds ratio

^b^ CI: Confidence interval

^c^ ALT: Alanine aminotransferase

^d^ ART: Antiretroviral therapy

^e^ NRTIs: Nucleoside reverse transcriptase inhibitors

^f^ HAART: Highly active antiretroviral therapy

^g^ IDU: Injecting drug use

^h^ CDC: Centres for Disease Control and Prevention

**Figure 1:**
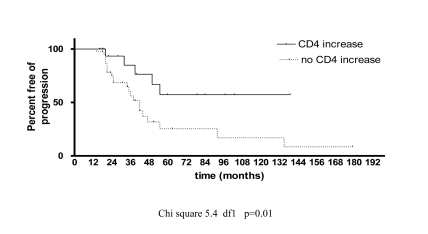
Analysis of life survival curve showing an increase of CD4 cells count between two biopsies and the correlation with slower LFP (Chi square 5.4 df1 P = 0.01)

No significant correlation was found between LFP and the presence of antiretroviral therapy at the first biopsy and also between the two biopsies. In the multivariate analysis, a decrease in CD4+ cell count between the two biopsies remained independently associated with LFP (Table 3). Analysis of life survival curves confirmed that an increase in CD4+ cell count between the two biopsies was significantly correlated with slower LFP (Figure 1). We determined the liver fibrosis progression rate per years (FPR) in the 27 patients who progressed: the median FPR was 0.51 (IQR 0.31-0.64). At this rate of fibrosis progression, the median expected time to cirrhosis was 11.7 years (IQR 9.3-19.3).

**Table 3:** Multivariate analysis (logistic regression) of factors associated with liver fibrosis progression (staging increase of one)

**Factors**	**OR a**	**IL, 95% CI**	**SL, 95% CI b**	***P *value**
**CD4+ decrease** (> 20%)	3.99	1.25	12.76	0.020
**ALT c** (> 150 IU/L)	3.10	0.85	11.31	0.087

^a^ OR: Adjusted odds ratio

^b^ CI: Confidence interval

^c^ ALT: Alanine aminotransferase

## 5. Discussion

A more rapid progression of liver disease in HIV/HCV co-infected patients is well documented in a large number of studies [10][11][12][24]. However, the majority of available studies on liver fibrosis progression used a single liver biopsy with an estimated duration of HCV infection to calculate FPR [17][18][19][24][25][26]. The calculation of FPR has been extensively applied but assumes linear progression through all stages of infection. This assumption has effectively no clear evidence base. In the current study, we evaluated LFP in patients who underwent two consecutive biopsies. The rate of progression of liver fibrosis in our cohort was variable: in fact, we observed rapid progression even in patients with a lower stage score at baseline (median FPR 0.51/year) with an expected time to cirrhosis of less than 12 years, whereas the majority of patients (53.5%) did not show progression. Thus, this study provides strong evidence that progression of liver fibrosis in HIV/HCV co-infected patients is not linear.

In addition, we demonstrated that, even in patients with mild liver disease at first biopsy (stage 0-1), progression of fibrosis could occur in a significant proportion (27.5%) in < 43 months. This is consistent with the findings of Sulkowski et al. who demonstrated that significant fibrosis progression occurred in 24% of 174 non-cirrhotic HIV/HCV patients, although no or minimal fibrosis was detected in 77% of these patients in the first liver biopsy [27]. The rate of LFP in HIV/HCV co-infected patients is clearly higher than that observed in HCV-monoinfected patients, where available data showed rates from 8% to 12% [28][29]. Different parameters have been previously identified as risk factors for severe liver fibrosis in HCV-positive patient cohorts: increased age, duration of HCV infection, high alcohol intake, high ALT serum levels, and high necroinflammatory index [15][19][21][30][31][32][33][34][35][36]. In our study, only high ALT serum levels were significantly associated to LFP in a univariate analysis. In the study by Sulkowski et al, levels of serum AST also play an important role in predicting future liver disease, leading to support of the use of this prognostic marker in HCV treatment decision algorithms [27].

Other factors related to HIV infection may be also involved in LFP. Several studies have previously demonstrated that a low CD4+ cell count is strongly associated with rapid progression of fibrosis in HIV/HCV co-infected patients [15][21][36][37]. Immunosuppression due to HIV could induce modifications of cytokine patterns in the liver towards a Th2 response, which has been demonstrated to be associated with advanced fibrosis in animal models [37][38][39]. Regarding the role of antiretroviral therapy, in a recent study, Brau et al. [26] showed that HIV/HCV co-infected patients with HIV RNA < 400 cp/mL exhibited FPR similar to HCV monoinfected patients (0.122/year vs. 0.128), whereas patients with detectable HIV RNA had a higher FPR (0.15/year). In this study, HIV RNA, and not CD4+ cell count, was the best predictor of liver fibrosis. In another recent study, Verma et al. [25] compared 85 HIV/HCV co-infected and 296 HCV-monoinfected patients during a period of 10 years, and demonstrated that patients taking HAART as the first antiretroviral regimen had an FPR similar to HCV-monoinfected subjects; HAART, but not CD4+ cell count or HIV RNA level, was independently related to liver fibrosis. It is necessary to emphasize that in these studies, a single value of CD4+ cell count and HIV RNA, corresponding to the date of liver biopsy, were considered in the analysis, whereas in our study we evaluated not a single value, but the trend of the CD4+ cell count and, when available, HIV RNA levels in the period between the two biopsies. In fact, we observed that a decrease of more than 20% in CD4+ cell count between the two biopsies was independently correlated to LFP. Additionally, in 20 patients who were taking HAART, the presence of detectable HIV RNA in more than 50% of determinations was related to a faster LFP, although without statistical significance, probably due to the small number of patients with available HIV RNA. Furthermore, we observed that an increase of more than 20% in CD4+ cell count between the two biopsies, which can be related to efficient antiretroviral therapy, was also significantly related to slower LFP. However, we demonstrated no association between LFP and the absence or specific type of antiretroviral therapy (HAART, combination of two NRTIs, or monotherapy), underlining that only effective therapy, which results in an increase in CD4+ cell count and HIV RNA suppression, is really relevant to reduce LFP.

Our findings that ALT levels and a decrease in CD4+ cell count, but not other parameters, were significantly correlated to LFP, might be due to the small number of enrolled patients and to the retrospective nature of the analysis; therefore, an increase in sample size might provide further information. A recent study on paired liver biopsy in HIV/HCV co-infected patients has demonstrated the efficacy of PEG-IFN therapy in reducing or stabilizing liver fibrosis, in comparison to untreated patients, who presented faster progression even in the absence of a sustained virological response [40]. In our study, we did not find a correlation with anti-HCV therapy, probably due to the fact that all our patients had been treated in the past, mostly with suboptimal schedules based on standard IFN monotherapy, or had prematurely discontinued treatment.

In conclusion, our data suggest that in HIV/HCV co-infected patients the introduction of effective antiretroviral therapy is also relevant to reduce LFP. Therefore, significant efforts should be made to obtain patients' adherence to antiretroviral therapy, in order to reduce the occurrence of viral resistance and guarantee a good immune-virological profile. Thus, early introduction of antiretroviral therapy should be considered in HIV/HCV co-infected patients, especially in the presence of contraindications to anti-HCV therapy or in patients that were non-responsive to previous antiviral treatments. The evidence of rapid progression in subjects with mild liver disease suggests the need for adequate screening and aggressive treatment of HCV infection even in this subset of patients.
